# Cytokines in Preterm Delivery: Proposal of a New Diagnostic Algorithm

**DOI:** 10.1155/2018/8073476

**Published:** 2018-04-08

**Authors:** Grzegorz Raba, Jacek Tabarkiewicz

**Affiliations:** ^1^Institute of Obstetric and Emergency Medicine, Faculty of Medicine, University of Rzeszow, Ul. Pigonia 6, 35-310 Rzeszów, Poland; ^2^Centre for Innovative Research in Medical and Natural Sciences', Faculty of Medicine, University of Rzeszow, Ul. Warzywna 1a, 35-959 Rzeszów, Poland; ^3^Department of Human Immunology, Faculty of Medicine, University of Rzeszow, Al. Mjr. W. Kopisto 2a, 35-310 Rzeszów, Poland

## Abstract

Predicting preterm delivery within 7 days is very important for the proper timing of glucocorticosteroid administration. If within 7 days after glucocorticosteroid administration, the delivery does not occur, it remains questionable if repeated glucocorticosteroid therapy results in improved infant respiratory function. Therefore, differentiation of preterm delivery from false preterm delivery is clinically significant. The aim of this study was to create a diagnostic algorithm to distinguish preterm delivery from false preterm delivery on the basis of concentrations of selected cytokines. The study group (*n* = 622) were patients hospitalized due to threatened preterm delivery. To assess the concentration of cytokines in the serum, we used a multiplex method, which allows simultaneous determination of 13 cytokines. The sets consist of the following cytokines: IGFBP-1, IGFBP-2, BDNF, L-Selectin, E-Selectin, ICAM-1, PECAM, VCAM-1, MIP-1d, MIP-3b, Eotaxin-1, Eotaxin-2, and BLC. In the study group, 67.8% patients had preterm delivery and 32.2% had false preterm delivery. Based on the analysis of cytokine concentrations, a classification tree to distinguish between preterm delivery and false preterm delivery was created. Our findings show the possibility of prediction of preterm delivery with the use of a classification and regression tree of selected cytokine concentration.

## 1. Introduction

Preterm birth (PTB), defined as birth prior to 37 weeks of gestation, affects 5–18% of pregnancies and is appraised to be the leading cause of neonatal death and the second cause of childhood death below the age of 5 years. The uterine overdistention, cervical diseases, breakdown of maternal-fetal tolerance, decidual senescence, vascular disorders, infections, and stress are the major mechanisms of the disease implicated in spontaneous preterm labor, but probably there are still unknown factors included in this pathology [1]. Physical activity has been theoretically related to PTB because it increases the release of catecholamines, for example, norepinephrine, which might stimulate myometrial activity. On the other hand, Mascio et al. showed that aerobic exercise can be safely performed by normal-weight women with singleton, uncomplicated gestations because this is not associated with an increased risk of preterm birth or with a reduction in mean gestational age at delivery [2]. The prevention of preterm birth by use of special diet or diet supplements, for example, omega-3 supplementation during pregnancy, does not reduce the incidence of PTB birth or improve neonatal outcome [3]. The goal of preterm delivery treatment is to reduce the effects of prematurity in a newborn. Based on an individual patient data, meta-analysis cerclage cannot currently be recommended for clinical use especially in twin pregnancies with a maternal short cervical length in the second trimester, because there are no statistically significant differences in primary and secondary outcomes [4]. According to data from The American Congress of Obstetricians and Gynecologists (ACOG), the factor that improves the prognosis for premature infants is not the delay of delivery but glucocorticosteroid administration within 7 days prior to delivery in order to improve respiratory function in the newborn [5]. On average, tocolytic treatment prolongs the duration of pregnancy by only 48 hours [6]. It is a short period, slightly increasing child maturity; however, this time period makes it possible to administer glucocorticosteroids to the mother, increasing the amount of surfactant in the newborn's alveoli and reducing the incidence and severity of respiratory distress syndrome (RDS) [7–10]. Gaining time to administer glucocorticosteroids to a woman at risk for preterm delivery is the main indication for tocolytic treatment. Improvement in lung respiratory function is maintained in a child for a period of around 7 days after administration of glucocorticosteroids to the mother [11, 12]. If there is no delivery within 7 days after steroid administration, it remains disputable whether repeated steroid therapy during the same pregnancy brings similar benefits of improvement in an infant's respiratory function [13].

Therefore, the decision to administer glucocorticosteroids in the treatment of women with threatened preterm delivery should be made if the probability of delivery in a period shorter than 7 days is thought to be small. This is difficult in clinical practice because currently there are no accurate diagnostic markers that allow for sensitive and specific differentiation of early preterm delivery from false preterm delivery. There are models of differential diagnosis of early preterm delivery and false preterm delivery based on the identification of risk factors [14]; however, in some cases, estimation of these risk factors is not possible. With the development of molecular biology techniques, great hopes lie in research on the identification of markers allowing for differentiation of early preterm delivery from false preterm delivery. It is indispensable for the proper timing of glucocorticosteroid application in a pregnant woman in order to improve respiratory function in a newborn. Varied etiopathogenesis of preterm delivery resulted from the fact that in the past decade identification of numerous cytokines and molecules connected with preterm delivery has not brought any significant progress in the differential diagnosis of early preterm delivery and false preterm delivery. The majority of the commercially available tests is based on the estimation of insulin-like growth factor binding protein (IGFBP-1) in combination with other molecules, for example, alpha-fetoprotein (AFP) or interleukin 6 (IL-6) [15–18]. Additionally, a majority of these immunoassays are used for detection and confirmation of fetal membrane rupture. Some methods, for example, fetal fibronectin testing in singleton gestations with threatened preterm labor, are associated with higher costs, but not with the prevention of preterm birth or improvement in perinatal outcome [19]. The complex pathogenesis of preterm labor authorizes the undertaking of trials to create multifactorial models that take into account the interactions and interrelationship of cytokines. In our study, we estimated serum concentrations of IGFBP-1 and twelve other markers and their usefulness for differentiation of preterm delivery from false preterm delivery. To the best of our knowledge, the majority of these markers is estimated on a large cohort of patients for the first time. The choice of these thirteen cytokines was justified by their potential role in inflammation processes involved in the pathogenesis of preterm delivery. IGFBP-1 and IGFBP-2 help to lengthen the half-life of circulating IGFs in all tissues and alter their interaction with cell surface receptors. Pregnant women who are in preterm labor with intact fetal membranes and who have a positive IGFBP-1 test result in cervical secretion have an increased risk of preterm delivery [20]. BDNF binds carboxypeptidase E (CPE), and the disruption of this binding has been proposed to cause the loss of sorting into dense-core vesicles. It may be involved in the pathogenesis of PD by modulating the activity of neurotransmitter receptors, including the alpha-7 nicotinic receptor. Nicotinic receptors are involved in direct and endocrine effects on the main processes of placental development [21]. L-Selectin is a molecule found on lymphocytes and the preimplantation embryo acting as a “homing receptor” for lymphocytes to enter secondary lymphoid tissues via high endothelial venules. It may be involved in the pathogenesis of PD by modulating the inflammation process [22]. E-Selectin during inflammation plays an important part in recruiting leukocytes to the site of injury, and its potential role in the pathogenesis of PD was described [22]. ICAM-1 is a type of intercellular adhesion molecule continuously present in low concentrations in the membranes of leukocytes and plays a role in spermatogenesis [23]. PECAM plays a key role in removing aged neutrophils from the body and is likely involved in leukocyte transmigration, which has a potential role in the pathogenesis of PD [24]. VCAM-1 mediates the adhesion of lymphocytes, monocytes, eosinophils, and basophils to the vascular endothelium and is responsible for fetal growth restriction and PD [25]. MIP-1d is chemotactic for neutrophils, monocytes, and lymphocytes and elicits its effects by binding to cell surface chemokine receptors. The role in the pathogenesis of PD was evaluated in recent studies [26]. MIP-3b may play a role in normal lymphocyte recirculation and homing. It also plays an important role in T cell and B cell migration to secondary lymphoid organs. The role in the pathogenesis of PD was evaluated in recent studies [26]. Eotaxin-1 selectively recruits eosinophils by inducing their chemotaxis, which has a potential role in the pathogenesis of PD by modulating the inflammation process [27]. Eotaxin-2 is strongly chemotactic for resting T lymphocytes and slightly chemotactic for neutrophils [27]. BLC is selectively chemotactic for B cells belonging to both the B-1 and B-2 subsets and elicits its effects by interacting with chemokine receptor CXCR5. The role in the pathogenesis of PD was evaluated in recent studies [26].

The aim of this study was to create a diagnostic algorithm differentiating preterm delivery from false preterm delivery on the basis of concentrations and interactions of chosen serum markers.

## 2. Materials and Methods

The study group consisted of 622 pregnant women hospitalized due to threatened preterm delivery. The consent to perform the study was obtained from the local bioethics committee. The diagnosis of threatened preterm delivery was based on clinical criteria by Czajka et al. [28]. We excluded patients

- With clinical signs of infection
- Who were treated with antibiotics up to 3 weeks before the study
- With placenta previa
- With placental abruption
- With multiple pregnancies
- With cervical incompetence

After the diagnosis was made, all patients were tested for concentrations of the analyzed markers, after which they were treated according to a single tocolytic procedure with the use of intravenous fenoterol infusion 0.05 mg/hour during 48 hours along with glucocorticosteroid therapy (2 doses of betamethasone 12 mg every 24 hours). According to the guidelines [6–8], after treatment, the patients were prepared for preterm birth. Peripheral blood samples were collected into a serum separating tube (Becton Dickinson, USA; Barricore tube with mechanical separator and lithium heparin for plasma separation) and after centrifugation with the use 1000 ×g for 5 min. at temperature 4°C and serum separation, samples were aliquoted and cryopreserved at −86°C. Concentration of selected markers was measured in the laboratory of RayBiotech, Norcross, GA, USA, with the use of Quantibody® Human Cytokine Array 1 Kit. Samples were transported in certified packages on dry ice. We determined concentrations of markers, which could be grouped into the following clusters:

1. Circulating proteins variably expressed during inflammatory phase: IGFBP-1, IGFBP-2, and BDNF
2. Adhesion molecules involved in leukocyte-endothelial transduction: L-Selectin, E-Selectin, ICAM-1, PECAM, and VCAM-1
3. Chemokines: MIP-1d, MIP-3b, Eotaxin-1, Eotaxin-2, and BLC

The results were compared with the final time of delivery. Preterm delivery was diagnosed in those pregnant women who had delivery within 7 days of administration of tocolytic treatment and glucocorticosteroid therapy. The final diagnosis of false preterm delivery depended on the stopping of contractions and occurrence of delivery after a period longer than 7 days since the completion of glucocorticosteroid treatment.

Discriminant analysis by standard and forward stepwise selection method included 13 proteins, of which changes in serum concentrations in women at risk for preterm delivery have been proved in many studies [2, 26, 29, 30]. We used Statistica 8.0 PL (StatSoft, Poland) with StatSoft Statistica neural networks package. The obtained results of analyses were used to create a model of differential diagnosis between preterm delivery and false preterm delivery, on the basis of a classification and regression tree [31].

## 3. Results

In the study group of 622 patients at threatened preterm delivery, 422 (67.8%) had preterm delivery in the period of a few hours to 7 days since the initiation of treatment. The remaining 200 (32.2%) had false preterm delivery. There were no statistical differences between women with preterm delivery and false preterm delivery, and the characteristics of both groups are summarized in Table 1.

**Figure 1:**
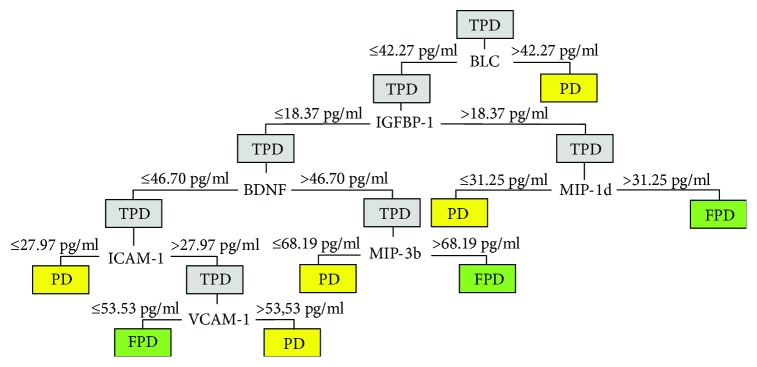
Diagnostic model to differentiate preterm delivery from false preterm delivery on the basis of biochemical markers concentrations. TPD: threatened preterm delivery; PD: preterm delivery; FPD: false preterm delivery.

We analyzed 13 markers which could be associated with preterm delivery IGFBP-1, IGFBP-2, BDNF, L-Selectin, E-Selectin, ICAM-1, PECAM, VCAM-1, MIP-1d, MIP-3b, Eotaxin-1, Eotaxin-2, and BLC, and we estimated their usefulness as markers for differentiation between threatened preterm delivery, preterm delivery, and false preterm delivery. The concentrations of the analyzed parameters are shown in Table 2. Due to the relatively low number of statistically significant differences in simple comparison between the two groups, we decided to perform more complex analysis. The results of the discriminant analysis are summarized in Tables 3 and 4. We found that 7 of the markers examined are significantly associated with preterm labor: IGFBP-1, BDNF, ICAM-1, VCAM-1, MIP-1d, MIP-3b, and BLC. We calculated cutoff points for these molecules with the use of ROC and chose those with the highest sensitivity and specificity. Based on predictive value and association with preterm delivery, we propose an algorithm for the differentiation of threatened preterm delivery, preterm delivery, and false preterm delivery as shown in Figure 1.

## 4. Discussion

The variation in cytokine correlation levels of many cytokines with preterm delivery [32, 33] suggests that the immune response is more complex and depends on many factors. Therefore, attempts to predict preterm delivery based on the concentrations of individual biochemical markers are not effective. In the present study, we developed a novel diagnostic model differentiating preterm delivery from false preterm delivery based on a classification tree.

Due to the multifactorial aetiology of preterm delivery, only joint interpretation of the concentrations of numerous groups of cytokines, taking into account their relative interactions, can be useful in predicting preterm delivery. The successes of the reductionist approach, based on a priori hypotheses, limit the possibilities of applying systemic information. New methodologies based on frameworks for simultaneous measurement of various factors seem to effectively displace the previously used univariate reductionist approach. This is particularly important in the context of complex systemic diseases such as preterm delivery. According to Laudanski et al. [34], reduction from general to particular can marginalize the role of other unexpected causative agents. A systemic approach, which allows for making hypotheses based on the multifactorial aetiology of the problem, seems to be better suited to research on preterm delivery. In certain cases, chronic immune reaction can lead to a weakened response, demonstrated by the reduction of concentrations of some cytokines. Therefore, in the diagnosis of preterm delivery, assessment of the relative proportions of individual biochemical markers is more important than searching for values of their cutoff thresholds as particular biochemical markers. In women with vaginal colonization with anaerobic bacteria at 18–22 weeks of gestation, distortion in the proportion between the concentration of IL-1*β* and IL-1Ra (receptor IL-1 antagonist) [35] has been demonstrated. An elevated level of IL-1*β* and reduced levels of IL-1Ra allowed for the identification of women at high risk of preterm delivery. Zhang et al. presented results proving that low VCAM-1 expression in the trophoblastic cell could be correlated to the pathogenesis and progression of gestational hypertension (GH) [25]. The gestational hypotension could be associated with preterm delivery, and we found that serum level of VCAM-1 is one of the factors in differentiating FPD and PD. Mešić Ðogić et al. and Eleje et al. showed that elevated concentration of IGFBP1 in cervical secretion were highly correlated with preterm labor [17]. In our study, we showed that also serum concentration of IGFBP1 could be used in algorithm differentiating PD, TPD, and FPD. The chemokines including CCL20 (MIP-3a) may play a role in the pathogenesis of preterm labor [27]. In our study, we found that other members of the chemokine family as MIP-3b and MIP-1d could be associated with preterm delivery. Identification of a single biomarker to predict spontaneous PD delivery poses a significant challenge due to the miscellany of clinical presentations and of the pathomechanisms involved in preterm birth. The presented multifactorial diagnostic model based on a classification tree makes verification of patients at threatened preterm delivery and prediction of delivery within 7 days simple and quick. This has a very important clinical significance as it helps in choosing the proper timing of glucocorticosteroid therapy in pregnant women in order to improve the respiratory function of the newborn. Application of glucocorticosteroids within 7 days before the onset of preterm delivery is currently the most effective method of improving perinatal results in preterm delivery [36]. Contrary to commercially available immunoassay tests, for example, ROM Plus® and AmniSure®, which are used for diagnosis of preterm membrane rupture (pPROM) [16], our algorithm could be used not only for confirmation of preterm delivery started as a subclinical pPROM but also for support of decision-making for the administration of glucocorticosteroids. The disadvantage of the presented system of differential diagnosis of preterm delivery and false preterm delivery is its novel character, as well as the small group covered by the study in a single-center research model. However, the results obtained in the period of 4 years in this group of 622 patients are encouraging and justify additional testing. The publication in this paper of a complete algorithm diagram for the diagnosis of preterm delivery means the author grants his permission for its use in both practice and for research purposes. Verification of the presented diagnostic model in other centers and in other populations would be of high educational value. Easy interpretation of results by means of the presented classification tree encourages such attempts.

## 5. Conclusions

Our findings show the possibility of predicting preterm delivery within 7 days using a classification and regression tree of concentrations of selected cytokines. Our algorithm is not restricted only for conditions like rupture of membranes but could also support the decision of treatment with glucocorticosteroids in patients who will deliver a baby within 7 days. Additionally, it could help to make the decision of treatment postponement in patients with a high probability that delivery would be later than 7 days from glucocorticosteroid administration.

## Figures and Tables

**Table 1:** Characteristic of the groups.

Parameter	Statistics	Preterm delivery	False preterm delivery	*p*
Maternal age (years)	Mean ± SEM	26.4	25.1	0.241
		5.22	5.62	
	Min–max	16–41	18–39	

Gravidity	Mean ± SEM	2	1.8	0.112
		1.04	1.18	
	Min–max	1–6	1–4	

Gestational age (weeks)	Mean ± SEM	31.1	28.3	0.068
		3.96	5.9	
	Min–max	22–36	23–36	

Fetal weight by USG examination	Mean ± SEM	1761	1642	0.136
		658	757	
	Min–max	507–2482	612–2420	

Maternal height (cm)	Mean ± SEM	162.5	164.2	0.231
		5.98	8.18	
	Min–max	150–175	153–181	

Maternal weight (kg)	Mean ± SEM	66.1	61.1	0.083
		12.18	15.5	
	Min–max	47–99	52–94	

Maternal weight before pregnancy (kg)	Mean ± SEM	57.9	54.4	0.061
		10.47	14.2	
	Min–max	42–90	45–79	

Vaginal pH	Mean ± SEM	5.4	5.3	0.133
		0.84	1.23	
	Min–max	4.2–7.2	4.2–5.9	

Blood hemoglobin (g/dl)	Mean ± SEM	11.6	11.2	0.074
		0.84	1.12	
	Min–max	9.8–13.8	10.2–14.1	

WBC (mm^3^)	Mean ± SEM	7271	6830	0.212
		638.55	594.8	
	Min–max	5340–26,000	4800–14,000	

**Table 2:** Comparison of concentration levels of the analyzed markers between preterm delivery and false preterm delivery groups.

	Preterm delivery		False preterm delivery		*p*
	Mean (pg/ml)	±SEM	Mean (pg/ml)	±SEM	
BDNF	49.41	4.10	31.87	2.33	0.004
BLC	45.28	5.69	22.75	2.42	0.005
Eotaxin-1	1.68	0.16	1.03	0.08	0.001
Eotaxin-2	9.37	0.08	9.21	1.36	0.607
E-Selectin	393.43	29.80	372.55	32.41	0.953
ICAM-1	502.77	42.30	459.14	33.20	0.699
IGFBP-1	177.97	41.57	144.57	33.35	0.001
IGFBP-2	429.72	65.63	424.00	34.55	0.695
L-Selectin	2369.2	318.13	2379.9	265.01	0.888
MIP-1d	25.01	6.63	27.79	6.10	0.092
MIP-3b	31.16	5.19	24.22	4.75	0.93
PECAM-1	27.94	12.03	31.78	17.66	0.248
VCAM-1	44.87	20.44	36.09	10.46	0.158

**Table 3:** Predictive values of selected markers marked with discriminant analysis by standard method.

Biochemical marker	Wilks's lambda	Partial Wilks's	*F*	*p*	Tolerance	1-toler
BDNF	0.636	0.891	5.733	0.021	0.851	0.148
BLC	0.601	0.943	2.840	0.099	0.796	0.204
Eotaxin	0.589	0.963	1.818	0.184	0.582	0.418
Eotaxin-2	0.581	0.976	1.143	0.290	0.678	0.322
E-Selectin	0.571	0.993	0.332	0.567	0.393	0.607
ICAM-1	0.577	0.983	0.815	0.371	0.661	0.339
IGFBP-1	0.623	0.911	4.603	0.037	0.724	0.276
IGFBP-2	0.567	0.100	0.001	0.995	0.512	0.487
L-Selectin	0.603	0.941	2.961	0.092	0.397	0.602
MIP-1d	0.567	0.100	0.004	0.951	0.691	0.309
MIP-3b	0.567	0.100	0.002	0.966	0.836	0.164
PECAM-1	0.657	0.863	7.453	0.009	0.779	0.221
VCAM-1	0.571	0.993	0.336	0.565	0.634	0.366

**Table 4:** Predictive value of selected markers marked with discriminant analysis by stepwise forward selection method.

Biochemical marker	Wilks's lambda	Partial Wilks's	*F*	*p*	Tolerance	1-toler
IGFBP-1	0.668	0.891	6.447	0.014	0.813	0.187
PECAM-1	0.687	0.867	8.103	0.006	0.864	0.136
Eotaxin-1	0.617	0.965	1.917	0.172	0.846	0.154
BDNF	0.657	0.907	5.460	0.023	0.938	0.062
BLC	0.641	0.930	3.987	0.050	0.887	0.113
L-Selectin	0.627	0.950	2.784	0.101	0.767	0.233
VCAM-1	0.611	0.976	1.327	0.254	0.867	0.133
Eotaxin-2	0.586	0.983	0.904	0.346	0.737	0.263
E-Selectin	0.590	0.990	0.501	0.482	0.437	0.563
ICAM-1	0.586	0.984	0.844	0.362	0.859	0.141
IGFBP-2	0.596	0.100	0.008	0.931	0.773	0.227
MIP-1d	0.594	0.997	0.136	0.713	0.830	0.170
MIP-3b	0.596	0.100	0.003	0.959	0.908	0.092

## Abbreviations

IGFBP-1: Insulin-like growth factor-binding protein 1
IGFBP-2: Insulin-like growth factor-binding protein 2
BDNF: Brain-derived neurotrophic factor
L-Selectin: CD62L
E-Selectin: CD62E
ICAM-1: Intercellular adhesion molecule 1
PECAM: Platelet endothelial cell adhesion molecule
VCAM-1: Vascular cell adhesion protein 1
MIP-1d: Chemokine (C-C motif) ligand 15 (CCL15)
MIP-3b: Macrophage inflammatory protein-3 (CCL19)
Eotaxin-1: C-C motif chemokine 11 (CCL11)
Eotaxin-2: Chemokine (C-C motif) ligand 24 (CCL24)
BLC: B lymphocyte chemoattractant.
